# Horizontal Transmission of the Heritable Protective Endosymbiont *Hamiltonella defensa* Depends on Titre and Haplotype

**DOI:** 10.3389/fmicb.2020.628755

**Published:** 2021-01-14

**Authors:** Heidi Kaech, Christoph Vorburger

**Affiliations:** ^1^Department Aquatic Ecology, Eawag (Swiss Federal Institute of Aquatic Science and Technology), Dübendorf, Switzerland; ^2^Department of Environmental Systems Science, Institute of Integrative Biology, Swiss Federal Institute of Technology in Zurich, Zurich, Switzerland

**Keywords:** *Aphis fabae*, *Hamiltonella defensa*, horizontal transmission, symbiont, titre

## Abstract

Secondary endosymbionts of aphids have an important ecological and evolutionary impact on their host, as they provide resistance to natural enemies but also reduce the host’s lifespan and reproduction. While secondary symbionts of aphids are faithfully transmitted from mother to offspring, they also have some capacity to be transmitted horizontally between aphids. Here we explore whether 11 isolates from 3 haplotypes of the secondary endosymbiont *Hamiltonella defensa* differ in their capacity for horizontal transmission. These isolates vary in the protection they provide against parasitoid wasps as well as the costs they inflict on their host, *Aphis fabae*. We simulated natural horizontal transmission through parasitoid wasps by stabbing aphids with a thin needle and assessed horizontal transmission success of the isolates from one shared donor clone into three different recipient clones. Specifically, we asked whether potentially costly isolates reaching high cell densities in aphid hosts are more readily transmitted through this route. This hypothesis was only partially supported. While transmissibility increased with titre for isolates from two haplotypes, isolates of the *H. defensa* haplotype 1 were transmitted with greater frequency than isolates of other haplotypes with comparable titres. Thus, it is not sufficient to be merely frequent—endosymbionts might have to evolve specific adaptations to transmit effectively between hosts.

## Introduction

Microbial symbionts are ubiquitous in insects. They often influence ecologically relevant traits and therefore the ecological niche of their hosts. For aphids, survival on a diet of amino-acid-poor plant sap is only possible because of a symbiosis with the γ-proteobacterium *Buchnera aphidic*ola, which lives in specialised tissues of their body (Baumann, 1995). Together, aphid and endosymbiont produce all the essential amino acids lacking from the aphid’s diet (Hansen and Moran, 2011). On their own, neither of the two organisms can survive, making this symbiosis a primary endosymbiosis. In addition to *B. aphidicola*, aphids can also harbour other bacterial endosymbionts. While these provide benefits to the aphid, such as protection against natural enemies (Oliver et al., 2003; Ferrari et al., 2004) or improved resistance to heat stress (Chen et al., 2000; Russell and Moran, 2006), they are not essential for the host’s survival. They are therefore referred to as secondary endosymbionts.

To be maintained in a host population, symbionts must transmit vertically from mother to offspring, horizontally between individuals, or both. Transmission mode is an evolutionary continuum that can change over time and impacts the relationship between host and symbiont (Ebert, 2013). At one end of the spectrum are the symbionts with strict and faithful vertical maternal transmission. Their dispersal depends completely on their host’s reproduction. Ideally, they limit the amount of energy that they extract from their host to the absolute minimum so that the host can produce a maximal number of offspring, that lead to the symbiont’s dispersal. Thus, the intimate association with their host’s reproduction drives vertically transmitted endosymbionts toward avirulence (Bull et al., 1991). At the other end of the spectrum, there are purely horizontally transmitted symbionts. As their dispersal does not depend on their host’s reproduction, they tend to extract enough energy from their host to maximise their horizontal transmission success—for example by increasing the number of infectious particles that can spread (Ewald, 1983). As a result of their lifestyle, purely horizontally transmitted symbionts tend to be more virulent than vertically transmitted symbionts (Fisher et al., 2017).

Transmission mode has strong implications for the ecology and coevolution of both host and symbiont (Chrostek et al., 2017). For example, competition between different horizontally transmitted symbiont strains can, but does not have to, lead to greater host exploitation through an escalation of virulence as both competitors try to obtain a greater share of the host’s resources (Frank, 1992; Nowak and May, 1994; McLean et al., 2018). Horizontal transmission via vectors is thought to be particularly prone to lead to high virulence in the host (Ewald, 1983) and costly endosymbionts can use occasional horizontal transmission in combination with vertical transmission to be maintained in competition with less virulent and purely vertically transmitted strains (Lipsitch et al., 1995).

The primary endosymbiont of aphids, *B. aphidicola*, relies purely on vertical transmission and lives intracellularly (Baumann, 1995). The situation of secondary endosymbionts like *Hamiltonella defensa* and *Regiella insecticola* (Moran et al., 2005) is more ambiguous. On one hand, secondary endosymbionts transmit vertically with nearly 100% efficiency in the lab (Fukatsu et al., 2000; Darby and Douglas, 2003; Vorburger et al., 2017), such that they are hardly ever lost from laboratory cultures. On the other hand, they have the potential to transmit horizontally, as they do not only occur intracellularly but also in the hemolymph (Fukatsu et al., 2000). In fact, occasional horizontal transmission is necessary to explain the strain distribution of secondary endosymbionts across aphids (Sandström et al., 2001; Russell et al., 2003; Henry et al., 2013). In nature, horizontal transmission of aphid facultative endosymbionts could occur through the sting of parasitoid wasps (Heath et al., 1999; Gehrer and Vorburger, 2012; Peccoud et al., 2014), through sexual transmission (Moran and Dunbar, 2006), or—based on evidence from other insects—via plant tissues and surface contamination (Darby and Douglas, 2003; Caspi-Fluger et al., 2012).

In this work, we investigated the horizontal transmission potential of 11 different isolates of the secondary endosymbiont *H. defensa* in black bean aphids (*Aphis fabae*). The symbiont provides protection against parasitoid wasps, but the strength of this protection varies greatly between different isolates (Cayetano et al., 2015). In addition, there are large differences in the cost and the titres that different isolates reach within their host (Cayetano et al., 2015). Interestingly, strength of protection and cost to the host are negatively, rather than positively, related across different isolates. This raises the question of how highly virulent isolates that provide limited benefits can persist in aphid populations.

We hypothesised that those *H. defensa* isolates that were found by Cayetano et al. (2015) to be highly protective and avirulent would rely primarily on vertical transmission, whereas isolates that were found to be costly but less protective would also depend on horizontal transmission. Costly isolates may extract more resources from their host to increase their abundance in the host’s hemolymph (Chong and Moran, 2016), which in turn may increase their chance to be successfully transmitted from one aphid to another by a parasitoid’s ovipositor. To test this hypothesis, we simulated horizontal transmission events using fine needles and correlated the titre of different *H. defensa* isolates with their horizontal transmission success.

## Materials and Methods

### Aphid Clones and *H. defensa* Isolates

For the horizontal transmission assay, we used 12 sublines of the *A. fabae* clone A06-407 as hemolymph donors. Clone A06-407 was originally free from secondary endosymbionts (“407H0”) and had been microinjected with 11 different *H. defensa* isolates from other *A. fabae* clones between 2008 and 2012 (Supplementary Table 1) to form sublines 407H15 to 407HAf6. Collection details of aphid clones and date of creation of sublines are provided in Supplementary Table 2 and Supplementary Table 1, respectively. Based on partial sequences of two bacterial housekeeping genes, *murE* and *accD*, the *H. defensa* isolates can be grouped into three haplotypes: Haplotype 1 comprising H76 and H101, haplotype 2 comprising H9, H28, H30, H323, H343, H402, and AF6, and haplotype 3 comprising H15 and H85 (Cayetano et al., 2015; Supplementary Table 1). The division into these three haplotypes has since been confirmed by sequencing additional genes (Youn Henry, personal communication). Costs and benefits of different *H. defensa* isolates were determined three years prior to this experiment by Cayetano et al. (2015). Both costs and benefits varied strongly between isolates, with highly protective isolate H76 causing no detectable reduction in lifetime offspring production and the less beneficial isolate H85 strongly reducing the amount of offspring. Costs of the other strains lay in between these two extremes (Supplementary Figure 1).

Hemolymph was transferred from each donor to three recipient *A. fabae* clones (A06-37, A06-405, and A06-407). The recipient clones were collected in summer 2006 in Europe (Supplementary Table 2) and are naturally free of any known secondary endosymbionts (Vorburger et al., 2009). Recipient names are abbreviated as 37H0, 405H0, and 407H0, with “H0” indicating the absence of the secondary endosymbiont *H. defensa*. We used three recipients to assess whether different genotypes differ in how readily they accept the *H. defensa* isolates, and we included clone A06-407 as a recipient to test whether *H. defensa* isolates have adapted to this clone since their introduction and are therefore more easily (re-)introduced to it. A fourth *H. defensa*-free clone, which is visibly distinguishable from all others due to a colour mutation, A08-28^H–^ (Supplementary Table 2), was included in the assays to check for potential horizontal transmission via plants (see Experimental Procedures below). Since collection or creation, all aphid clones and sublines were maintained in a clone bank on broad bean (*Vicia faba*) seedlings and under conditions that ensured clonal reproduction (18–20°C, 16 h photoperiod).

### Experimental Procedures

Subadult aphids (nymphs) of all three recipient clones were stabbed with fine pins contaminated by hemolymph from each of the 11 *H. defensa*-infected sublines of clone A06-407 as well as hemolymph from 407H0 as a symbiont-free control. All 36 donor-recipient combinations were replicated 5 times, with each replicate consisting of a batch of 10 stabbed nymphs. Transmission success was determined by testing the offspring of stabbed individuals for the presence of *H. defensa*. Therefore, we only counted horizontal transmissions as successful if they led to new heritable infections.

To produce enough aphids for the experiment, five adults of each aphid clone or subline were split off from the laboratory stocks and bred up on *V. faba* seedlings for two generations. Adults used to found the third generation were allowed to reproduce on new plants for 24 h, then they were frozen at −20°C and used to confirm the presence/absence of *H. defensa* and the correct *H. defensa* haplotype at the start of the experiment (see section Molecular Methods below).

To produce the aphids acting as hemolymph donors, 10 adults of the second generation of each subline were placed on a new bean plant, allowed to reproduce for 24 h and then transferred to a next plant. This was repeated over 5 days to produce five consecutive batches of offspring. Offspring were reared for 15 days until they reached adulthood, at which point they were used as donors in the transmission assays.

To produce the aphids acting as hemolymph recipients, each of the three recipient clones was reared on 12 separate plants for two generations. We then took six adults from each plant, placed them on a new plant to reproduce for 24 h, before moving them on to another new plant. Again, this was repeated over 5 days to produce five batches of offspring. Offspring were used as recipients in the transmission assays as 3-day old nymphs.

Manual transfection of hemolymph from donor to recipient took place over five consecutive days. For every combination of donor and recipient, one 15-day old donor aphid was used to infect 10 three-day-old recipient nymphs. The donor was mounted with double-sided tape on a glass slide and stung with a fine stainless steel needle (Minutien pins, 0.1 mm diameter, Fine Science Tools GMBH), which was then inserted briefly into a recipient nymph. This double stabbing procedure was repeated for each of the 10 nymphs. Before use, the needles had been sanitised by soaking in 70% ethanol for 5 min, but they were not cleaned between successive stabs of the same donor-recipient clone combination. Aphids were under CO_2_-anesthesia during the procedure. Except for the first day, the donors were frozen after use for later reference. On each day we collected a pool of three donors per subline, which was frozen at −20°C until DNA extraction and subsequent qPCR for estimation of *H. defensa* titres. A graphical overview of the experimental procedures is provided in Supplementary Table 3.

After transfections, the 10 nymphs of each donor-recipient combination were transferred to a ventilated insect breeding dish (Ø 5 cm), which contained a broad bean leaf disc (Ø 4 cm) on 1% agar, and were maintained at 21°C and a 16 h photoperiod. To test whether rearing all 10 nymphs on the same leaf disc might allow between-nymph transmission of *H. defensa* via the leaf tissue, we also added two three-day-old nymphs of the *H. defensa*-free colour-mutant clone A08-28^H–^ as sentinels to each disc. The number of surviving recipients in each disc was scored six days after transfection, when aphids approached adulthood. Either four (batch 1) or three (batches 2–5) survivors were transferred individually to new leaf discs to be reared until their offspring reliably indicated status of infection. Preliminary experiments had established this to be the case at approximately 13-days old, a finding which is corroborated by literature (Chen and Purcell, 1997). The two sentinel aphids of each leaf disc were raised together on a new leaf disc, reared for 7 days and then frozen at −20°C until DNA extraction.

Three days after isolating the surviving recipients on separate leaf discs, the number of offspring produced by each survivor was noted (time point t1) and the offspring discarded. After another 4 days, the survivors were moved to new leaf discs and the offspring on the old leaf disc were counted (time point t2) and discarded. After reproducing for 2 days on the new leaf discs, the survivors were removed from the leaf disc and frozen at −20°C. After an additional 5 days, a pool of three offspring of each survivor was harvested for diagnostic PCR for *H. defensa* to test whether their stabbed mothers had acquired and passed on the symbiont. The three nymphs from each leaf disc were collected into collection microtubes of the DNeasy 96 Blood & Tissue kit (Qiagen AG, Hombrechtikon, Switzerland) to which two sterile glass beads were added.

In the second batch, offspring of one A06-407 recipient exposed to hemolymph from donor 407H0 tested positive for *H. defensa*, which is only explicable by contamination. To identify its source, we tested all available H0 donors (batches 2–5) and the mothers of all A06-407 recipients for presence/absence of *H. defensa*. This revealed that the recipient culture was clean, but the donor culture of 407H0 had been contaminated with *H. defensa*-infected individuals. Four of the twelve H0 donors used in batches 2–5 were found to have carried *H. defensa*. We discarded their data as well as all data from H0 donors in batch 1, since these donors had not been saved and could therefore not be checked retrospectively.

### Molecular Methods

DNA was extracted from aphids using a “salting out” protocol (Sunnucks and Hales, 1996) for checking the identity of aphids used to set up the experiment. For estimating *H. defensa* titres in donor lines and for assessing whether offspring produced by recipient aphids carried *H. defensa* we used the Qiagen DNeasy 96 Blood & Tissue kit (Qiagen AG, Hombrechtikon, Switzerland). Extraction success was verified by amplifying part of the 16S rRNA gene of *B. aphidicola*, the obligate endosymbiont present in all aphids, using specific primers. The presence/absence of *H. defensa* was also determined by diagnostic PCR with specific primers for the same gene. Primers and cycling conditions are detailed in Supplementary Table 4. Amplicons were run and visualised either by agarose gel electrophoresis or by capillary electrophoresis on a QIAxcel Advanced System (Qiagen AG, Hombrechtikon, Switzerland). To identify *H. defensa* haplotypes, we amplified *murE* and *accD* gene fragments (primers and cycling conditions in Supplementary Table 4) for Sanger sequencing by a commercial provider (GATC Biotech AG, Köln, Germany).

The density of *H. defensa* in the 11 donor lines was estimated from five replicate pools of three individuals (one from each batch), using TaqMan real-time quantitative PCR as described by Schmid et al. (2012). The ratio of *H. defensa*’s *dnaK* and the aphid’s *EF1*α gene served as a proxy for *H. defensa* titre. The 20 μl reactions were run in triplicates on a LightCycler 480 (Roche) and gene copy number was estimated based on a standard curve produced with serial dilutions of a synthetic standard provided by Microsynth AG (Balgach, Switzerland). For analysis, we used the 2nd Derivative Absolute Quantification approach in LightCycler 480 SW 1.5.1. Two replicates were excluded because we failed to harvest three live adults for DNA extraction (batch 3 of 407H323 and batch 4 of 407H85).

### Statistical Analysis

Statistical analyses were performed in RStudio v1.2.5033 (RStudio Team, 2020) and R v3.6.3 (R Core Team, 2018) using the packages reshape2 v1.4.4 (Wickham, 2007) and dplyr v0.8.5 (Wickham et al., 2020) for data wrangling, lme4 v1.1-23 (Bates et al., 2015) for linear mixed models and generalised linear mixed models, DHARMa v0.3.3.0 (Hartig, 2020) for residual analysis and ggplot2 v3.3.2 (Wickham, 2016) for producing figures. *Post-hoc* tests were performed with multcomp v1.4-13 (Hothorn et al., 2008). Anova-like tables of random effect terms of linear mixed models using likelihood ratio tests were produced with lmerTest v3.1-2 (Kuznetsova et al., 2017).

To compare *H. defensa* titres among different isolates and haplotypes, the ratio of *H. defensa dnaK* to *A. fabae EF1*α copy numbers was log-transformed and used in a linear mixed model with haplotype as fixed and *H. defensa* isolate as random effect. Since haplotype did not significantly influence titre, we also ran a linear model with just *H. defensa* isolate as predictor. For further analyses, the titre of each isolate was averaged over all five batches. For models with transmission rate as response variable, we only included donors that carried *H. defensa* (i.e., excluding 407H0). We used a generalised linear mixed model (GLMM) with binomial errors, expressing transmission rate as aggregated binomial data juxtaposing number of infected to number of uninfected individuals per treatment and batch. We treated average titre, recipient clone and donor haplotype as fixed effects and included the interactions between recipient and titre and the interaction between recipient and haplotype. Donor identity and batch were treated as random intercepts. We assumed there to be no interaction of titre and haplotype. The data indicates that this is true for haplotype 2 and 3, and it is impossible to determine a reliable estimate of the slope for haplotype 1 since both isolates of this haplotype (H76 and H101) have near-identical transmission rates and titre. To allow the model to converge, we used the Bobyqa optimiser with a set maximum of 200,000 iterations. Uniformity of residuals was confirmed using the package DHARMa (Hartig, 2020), which uses a simulation-based approach, and the model was checked for overdispersion.

To analyse survival of stabbed nymphs, we used a linear mixed effects model on the arcsine-square-root-transformed proportion of surviving nymphs. Average titre, recipient clone and donor haplotype (including *H. defensa*-free donors as a fourth “haplotype”) as well as the interaction between recipient and titre and recipient and haplotype were treated as fixed effects, and batch and donor were random intercepts.

The fecundity of survivors during the first week of their adult life was analysed using a GLMM with negative binomial errors. We excluded aphids that had not survived until time point t2 from the data set as their infection status could not be reliably assessed. Recipient clone and infection status (“control,” donor not infected with *H. defensa*; “exposed,” donor infected with *H. defensa* but the infection was not transmitted to the survivor’s offspring; “infected,” the infection was transmitted to the survivor’s offspring) were treated as fixed effects, and batch and donor as random intercepts. In an additional GLMM fecundity of successfully infected aphids was compared. Donor was treated as a fixed effect and batch as a random intercept.

## Results

**FIGURE 1:**
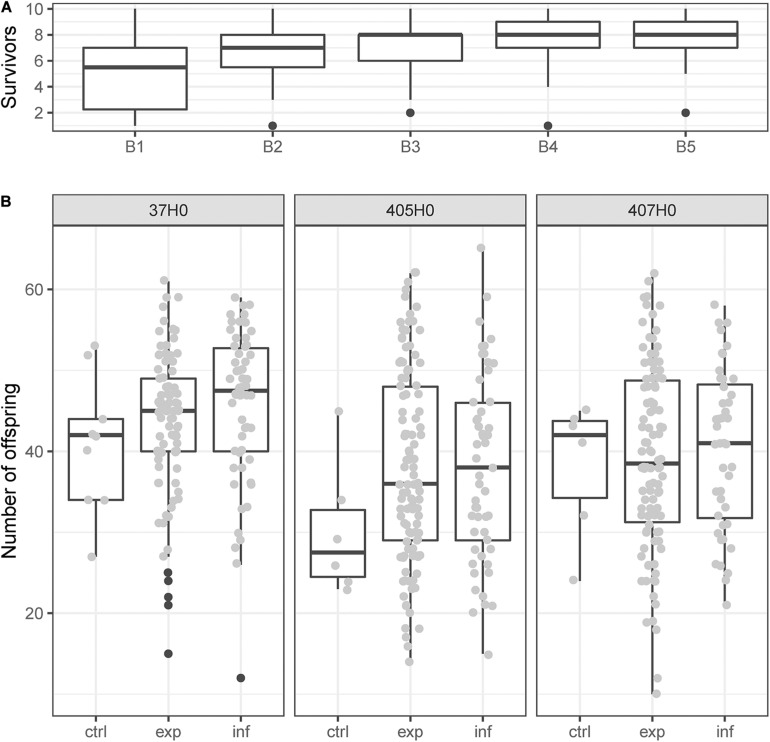
**(A)** The number of nymphs out of ten that survived being stung with a hemolymph-contaminated needle increased while processing the five consecutive experimental batches B1 to B5. **(B)** Reproduction of young adults of clones 37H0, 405H0, or 407H0 during 1 week. Recipients had been stung with a needle contaminated with hemolymph from a donor without *H. defensa* (control, “ctrl”) or with hemolymph from a donor with *H. defensa*. Recipients that had been exposed to potentially infectious hemolymph could either reject the infection (“exp”) or succumb to it (“inf”). Black dots indicate boxplot outliers.

Of 1,800 recipients (5 times 10 nymphs for each of the 36 donor-recipient combinations) that were stabbed with hemolymph-contaminated needles, 1,209 (67%) survived the transfection. Survival was different between batches (χ^2^ = 18.28, *df* = 1, *p* < 0.001) but not between different isolates of *H. defensa* (χ^2^ = 0.01, *df* = 1, *p* = 0.943), and did not depend on titre [*F*_(1, 7)_ = 0.08, *p* = 0.786] or haplotype [*F*_(3, 9)_ = 0.12, *p* = 0.943] of the *H. defensa* isolate that was transfected. Also non-significant were the effects of the recipient clone [*F*_(2, 147)_ = 1.05, *p* = 0.353], the interaction between titre and recipient [*F*_(2, 147)_ = 2.16, *p* = 0.119] and the interaction between recipient and haplotype [*F*_(6, 147)_ = 0.79, *p* = 0.583]. The significant influence of batch on survival was likely a result of improving skill and routine of the experimenter (Figure 1A).

A maximum of four (in batch 1) or three (in batches 2–5) nymphs of each treatment that survived the transfection were kept until their offspring would reliably indicate infection with *H. defensa*. We worked with 479 survivors instead of 576, since we did not always have as many nymphs survive as desired (*n* = 50), survivors died before their offspring would reliably indicate *H. defensa* infection (*n* = 28), and we discarded recipients stung with 407H0 donors that were not confirmed to be free of contamination with *H. defensa* (*n* = 19).

On average, survivors produced 40.5 ± 0.5 nymphs during their first week of reproduction. Fecundity in early adult life varied significantly among the three recipient clones (χ^2^ = 36.98, *df* = 2, *p* < 0.001), with clone 37H0 producing more offspring (Figure 1B), but did not depend on the recipient’s infection status (χ^2^ = 5.10, *df* = 2, *p* = 0.078). Thus the number of offspring produced in early adult life was similar between aphids that had acquired an infection with *H. defensa*, those that had rejected an infection and those that were not exposed to infectious hemolymph (Figure 1B). Among aphids that had been successfully infected through the transfection, fecundity was not significantly influenced by the infecting isolate’s identity (χ^2^ = 10.85, *df* = 10, *p* = 0.37).

**FIGURE 2:**
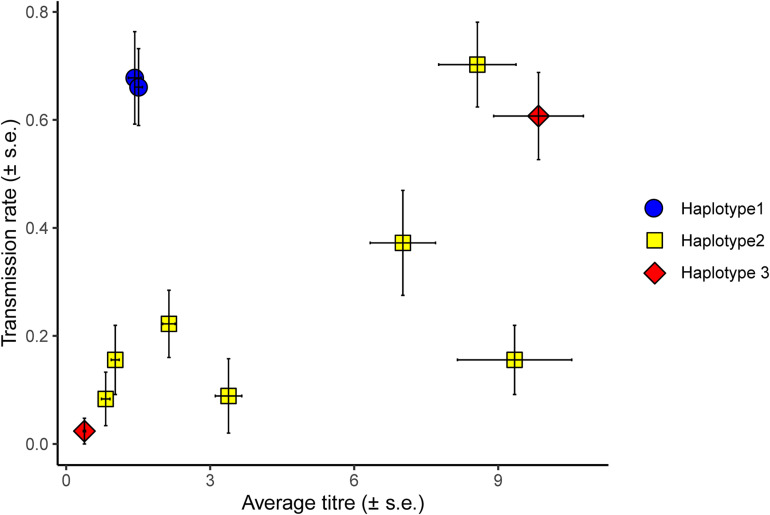
Average titre of different *H. defensa* isolates in donor aphids plotted against the average transmission rate of the isolate. Transmission rate corresponds to the number of recipients out of three or four in which a *H. defensa* isolate successfully established after horizontal transmission, i.e., was propagated to the recipient’s offspring. In this figure, transmission rate is averaged over three different recipients (37H0, 405H0, and 407H0) and five experimental batches. Error bars indicate the standard error and the combination of colours and symbols indicate the haplotype of the *H. defensa* isolate (blue circle = haplotype 1, yellow square = haplotype 2, red diamond = haplotype 3).

Horizontal transmission of *H. defensa* isolates from donor to recipients was considered successful if the symbiont managed to transmit to the recipient’s offspring. Of the 479 survivors of the transfection, 458 had been stabbed with hemolymph of *H. defensa*-infected donors. Of these, 156 (34%) produced *H. defensa*-positive offspring. These are indeed infections acquired from the stab and not infections acquired secondarily from other nymphs feeding on the same leaf disc, because none of the 177 sentinel aphids we tested were positive for *H. defensa*. Horizontal transmission success varied between different *H. defensa* isolates and batches, which was reflected in a highly significant random effect of *H. defensa* isolate and a significant effect of batch (Table 1 and Figure 2). Both haplotype and titre significantly affected the transmission rate of an isolate (Table 1). Endosymbiont titre—expressed as the ratio of *H. defensa dnaK* to *A. fabae EF1*α copy numbers—varied significantly among different *H. defensa* isolates [*F*_(10, 42)_ = 39.86, *p* < 0.001] but not between haplotypes [*F*_(2, 8)_ = 0.38, *p* = 0.697] (Supplementary Figure 2 and Figure 2).

**TABLE 1:** Results of a generalised linear mixed effects model for the transmission rate of different *H. defensa* isolates to different recipients (37H0, 405H0, and 407H0).

	**Effect**	**LR χ^2^**	**df**	***p*-value**
Random:	Donor	24.48	1	**<0**.**001**
	Batch	4.29	1	**0**.**038**
Fixed:	Titre of *H. defensa* isolate	8.93	1	**0**.**003**
	Haplotype of *H. defensa* isolate	11.18	2	**0**.**003**
	Recipient clone	3.52	2	0.172
	Titre: recipient	8.81	2	**0**.**012**
	Recipient: haplotype	10.26	4	**0**.**036**

*Model predictors were recipient, average titre that an isolate reaches in the donor aphid and haplotype of the isolate (haplotypes 1, 2, and 3). The aphid subline acting as donor during horizontal transmission (“donor”) and experimental batch were treated as a random effect. Bold indicates significance (p < 0.05).*

**FIGURE 3:**
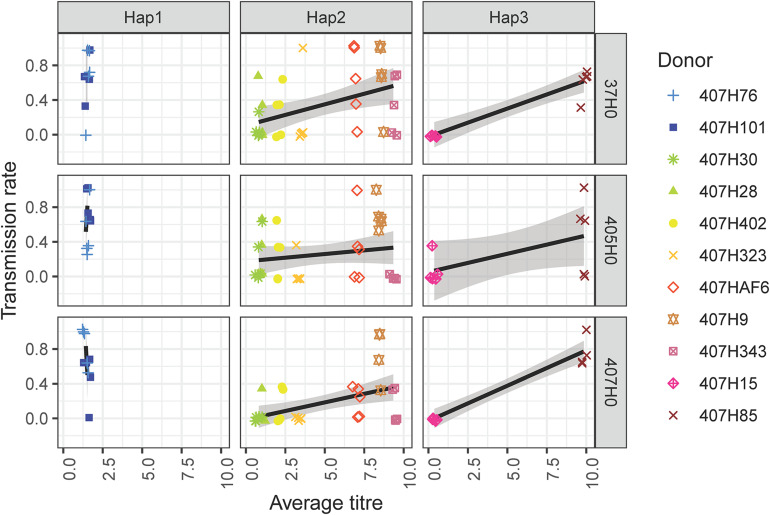
Eleven *H. defensa* isolates were horizontally transfected from donor aphids (clone A06-407) to recipients (37H0, 405H0, and 407H0). Transmission rate corresponds to the number of recipients out of three or four in which a *H. defensa* isolate successfully established after horizontal transmission, i.e., was propagated to the recipient’s offspring. The combination of colours and symbols indicates the identity of the different isolates (“donor”). Transmission rate is plotted against average titre of the isolate on the x-axis. To allow that data points with similar coordinates can be discerned, a jitter of 0.1 has been applied to both the x- and the y-axis.

In general, *H. defensa* isolates that reached a high titre in the donor were more frequently transmitted (Figures 2, 3): The seven isolates of haplotype 2 varied widely in their titres, and their horizontal transmission rates increased with titre. The two isolates of haplotype 3 reached the lowest and one of the highest titres, and the high-density isolate H85 was transmitted much more frequently than the low-density isolate H15. However, isolates H101 and H76 of haplotype 1, which both had comparably low titres, were much more successful at horizontal transmission than isolates from other haplotypes with similar titre (Figure 2).

Recipient clones did not differ in how frequently they acquired *H. defensa* (Table 1). Hence there was no evidence that *H. defensa* established more readily in the same genetic background as it was maintained in for several years. However, since recipient 405H0 was less susceptible to infection with high-titre isolates than 407H0 and 37H0 (Figure 3), there was a significant interaction between titre and recipient (Table 1). Also, isolates of haplotype 2 transmitted somewhat less frequently to recipient 407H0 (Figure 3), leading to a marginally significant interaction between recipient and haplotype (Table 1).

Recipients of the first experimental batch showed rather poor and highly variable survival (Figure 1A). To verify that the horizontal transmission results were not overly influenced by this batch, we repeated the analysis with a reduced dataset only containing batches 2–5. The random batch effect was indeed no longer significant in this analysis, but all the significant fixed effect remained significant (Supplementary Table 5), and the estimates of horizontal transmission rates remained nearly unchanged (Supplementary Figure 3).

## Discussion

This study was motivated by the somewhat counterintuitive observation in Cayetano et al. (2015) that costs and benefits of different *H. defensa* isolates to the aphid host are negatively correlated. Infection with *H. defensa* generally shortens *A. fabae*’s lifespan and thereby lifetime reproduction in the absence of parasitoids (Vorburger and Gouskov, 2011; Vorburger et al., 2013), yet the magnitude of this cost is very low for the most strongly protective isolates and higher for isolates providing less protection against parasitoids (Cayetano et al., 2015). We hypothesised that costly isolates of *H. defensa* may persist in host populations because they gain some fitness from horizontal transmission, for example via parasitoid wasps (Gehrer and Vorburger, 2012), aided by a high density in the host. Isolates that reach a higher titre in the host’s hemolymph might increase their horizontal transmission rate as the wasp’s ovipositor is more likely to be contaminated with enough bacteria to establish an infection in a new host.

Our results supported this hypothesis only partially. We confirmed that isolates differ in their titre, we demonstrated that simple stabs with symbiont-contaminated needles did indeed result in horizontal transmission, and we found that—within *H. defensa* haplotypes—higher titre was connected to increased transmission rate. Yet, our results also show that transmission rate depended strongly on *H. defensa* haplotype. Despite their low titre, isolates H76 and H101 of haplotype 1 transmitted considerably better than isolates of haplotype 2 and 3 with similarly low titres. Clearly it is not enough to enter the host in sufficient numbers—*H. defensa* also needs to overcome further hurdles on the way to establishing an infection, and the two isolates of haplotype 1 appear to be better suited to that task than others. Such among-strain variation in transmission success is also known from other bacteria, e.g., *Borrelia* in ticks (Tonetti et al., 2015), *Vibrio* in squid (Bongrand and Ruby, 2019), or potentially, *Wolbachia* in leaf cutter ants (Tolley et al., 2019).

Currently, there is no known mechanistic basis of the observed differences in *H. defensa* transmission success. It is possible that *H. defensa* isolates of haplotype 1 are better at evading the host’s immune system. Given that immune activation is a costly response (Moret and Schmid-Hempel, 2000; Zuk and Stoehr, 2002), their limited fitness effect on the host would also be consistent with them evading recognition by the immune system. Further studies will be needed to detect how isolates of haplotype 1 interact differently with the host than isolates of haplotype 2 and 3.

The assumption that costly *H. defensa* isolates with limited benefit for the host are maintained in the population by a greater disposition for horizontal transmission must therefore be rejected for our system. Instead, isolates of haplotype 1 seem omnipotent: They provide near-complete protection from parasitoid wasps (Cayetano et al., 2015), they hardly impair their host’s offspring production and thus maximise their vertical transmission potential (Cayetano et al., 2015), and they are efficient in transmitting horizontally. This leaves us with a problem: Why do these isolates not go to fixation in the field? Likely, isolates H76 and H101 have hidden costs, or costly isolates have hitherto unknown benefits. It is conceivable that, similar to the interaction between host and *Wolbachia*, factors such as environmental temperature influence bacterial titre in the field, resulting in increase or decrease of costs, or even loss of the symbiont under certain conditions [reviewed in López-Madrigal and Duarte (2019)]. Both aphid and whitefly endosymbionts have been shown to influence the interaction of host and plants and to change dietary breadth (Su et al., 2015; Wagner et al., 2015). Similarly, yet unexplored interactions of *H. defensa* with host plants might lead to costs or benefits in certain habitats.

The three different aphid clones acting as recipients in our experiment did not differ in their susceptibility to infection by *H. defensa*. Two of the clones were novel hosts for the *H. defensa* isolates, while they had been associated with clone A06-407 for at least 150 host generations prior to the experiment. Despite this long lasting association—longer in fact than any that we would find in the field, where symbiont-host genotype associations are re-shuffled when *A. fabae* reproduces sexually in autumn—infection success after horizontal transmission was the same in the “known” aphid genotype as in the two “novel” genotypes. On one hand, this result might be influenced by the way our clonal aphid stocks are propagated. At every generation, a small number of adult aphids are used to found the next generation. The genetic drift resulting from these repeated bottlenecks may have restricted the potential for host-symbiont co-adaptation. On the other hand, this result could reflect the fact that outside of the laboratory, *A. fabae* generally reproduces by cyclical parthenogenesis (Sandrock et al., 2011). The yearly bout of sexual recombination of host genotypes may exert selection on heritable endosymbionts to be “good mixers” that can survive in any genetic background.

In addition to the transport by parasitoid wasps acting as vectors (Gehrer and Vorburger, 2012), aphid endosymbionts can also be transmitted horizontally by sex (Moran and Dunbar, 2006), or potentially via physical contact or via the host plant (Darby and Douglas, 2003; Pons et al., 2019). The latter was a potential problem for our experiment, because 10 aphids that had been exposed to hemolymph from one donor were placed on the same leaf disc. Therefore, they fed on the same leaf during the first 6 days in which the infections established in the aphids. To detect whether *H. defensa* transmitted through the leaf, we placed uninfected sentinel aphids on the same leaf disc. In no case did we detect successful transmission of *H. defensa* to the sentinel aphids. It is unlikely that the sentinel clone A08-28 is resistant to infection with *H. defensa* as it was found in nature carrying a natural infection with *H. defensa*, from which it was cured in the laboratory to generate line A08-28^H^**^–^**. It is more likely that *H. defensa* does not transmit via the leaf, or that titres of *H. defensa* in newly infected individuals were too low to allow transmission through the leaf or through physical contact. Even though we cannot exclude that plant-mediated horizontal transmission might play a role in natural settings, it did not influence the observed horizontal transmission rate in our experiment (Ewald, 1987).

Interestingly, we did not observe any negative effects of newly acquired infections with *H. defensa* in terms of aphid survival directly after transfection nor in offspring production of survivors of the transfection. Generally, the survival rate in our experiment is comparable to the survival rate achieved in Niepoth et al. (2018), where *H. defensa* was transmitted via microinjection from infected pea aphid donors to pea aphid recipients that had been cured from their *H. defensa* infections. In our experiment, survival did not depend on whether hemolymph with or without *H. defensa* was transfected and did not vary significantly between different isolates of *H. defensa*. This stands in contrast to the results of Niepoth et al. (2018), where one of the two *H. defensa* isolates significantly reduced survival of all three recipients.

Previous studies have shown that infection with some *H. defensa* isolates decreases the host’s lifespan and lifetime offspring production (Vorburger and Gouskov, 2011; Cayetano et al., 2015; Niepoth et al., 2018). One reason that we did not detect such a difference may be that several isolates only rarely succeeded in horizontal transmission, which decreased the power to detect a significant difference in offspring production between recipients that did get infected by *H. defensa* and those that did not. More importantly, though, the time over which we quantified reproduction (1 week) was likely too short to detect the reproductive costs imposed by *H. defensa*, which are typically late-acting costs, mediated by curtailed lifespan rather than a reduced daily fecundity (Vorburger and Gouskov, 2011). We would expect costs on offspring production to be visible with increased sample size and by assessing lifetime reproduction instead of just reproduction during the first week of adult life.

## Conclusion

In conclusion, our experimental simulation of a known horizontal transmission route showed that both titre and haplotype of a *H. defensa* isolate determine its horizontal transmission success. This casts doubt on our initial assumption that costly isolates providing limited benefit to the host are maintained through an increased disposition for horizontal transmission, since two strongly protective and nearly avirulent isolates also showed frequent horizontal transmission. Further studies will be required to understand why such seemingly omnipotent isolates do not dominate in aphid populations.

## Data Availability Statement

The data has been provided to Dryad Digital Repository and can be accessed at https://doi.org/10.5061/dryad.s4mw6m95n.

## Author Contributions

HK collected the data and wrote the first draft of the manuscript. Both authors designed the study, contributed to data analysis, contributed to and commented subsequent versions, and approved the final manuscript.

## Conflict of Interest

The authors declare that the research was conducted in the absence of any commercial or financial relationships that could be construed as a potential conflict of interest.
