# Supplementary material for: Horizontal Transmission of the Heritable Protective Endosymbiont Hamiltonella defensa Depends on Titre and Haplotype
Source: Front Microbiol. 2021 Jan 14;11:628755. doi: 10.3389/fmicb.2020.628755 (PMC7840887; doi:10.3389/fmicb.2020.628755)
Supplement: Supplementary file 1 [file Data_Sheet_1.PDF]

Supplementary material

Supplementary figures

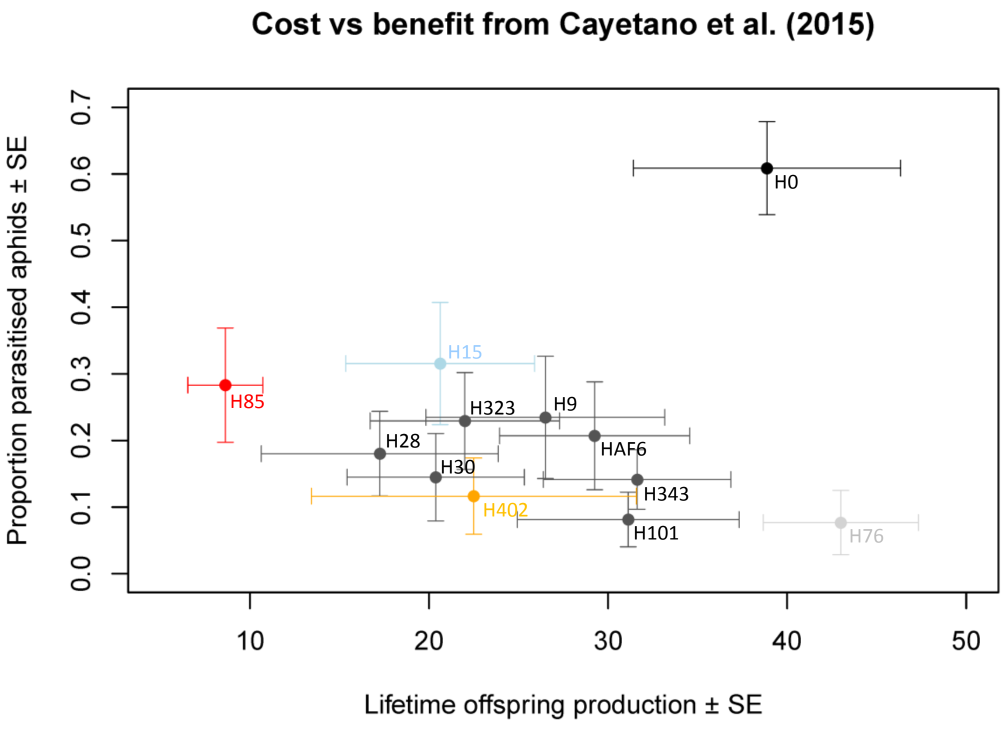

Supplementary Figure 1 – Figure adapted from Cayetano et al. (2015): Impact of infection with different *H. defensa* isolates on offspring production (cost) and parasitation success of the parasitoid wasp *Lysiphlebus fabarum* (benefit) in aphid clone A06-407. The aphid clone is uninfected (H0) or infected with different *H. defensa* isolates). For clarity, some isolates are marked by different colors: The highly protective and avirulent isolate H76 (light grey) of haplotype 1, one isolate of haplotype 2 (H402, orange), and the two isolates of haplotype 3 (H15 in light blue, and the very costly H85 in red). Error bars depict the standard error.

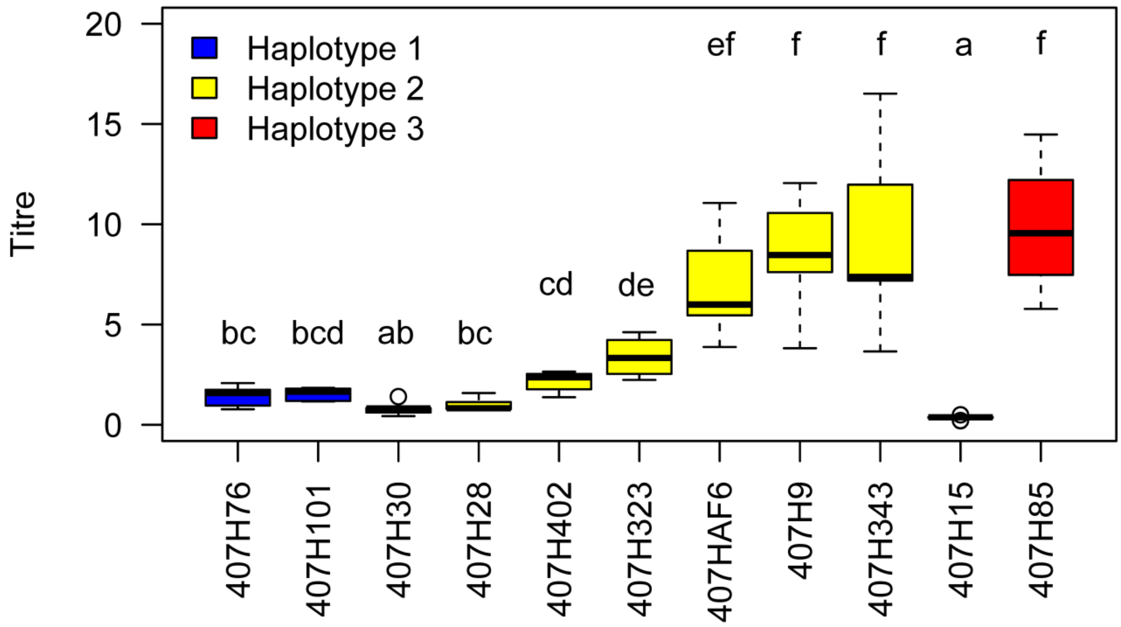

Supplementary Figure 2 – Titre of *H. defensa*, expressed as the ratio of gene counts from the two housekeeping genes *dnaK* (*H. defensa*) and *EF1α* (*A. fabae*), was measured from pools of three aphids for each of the five batches processed in the experiment using qPCR. The *H. defensa* isolates are coloured according to haplotype (blue = haplotype 1, yellow = haplotype 2, red = haplotype 3). Different letters indicate significant differences between titres.

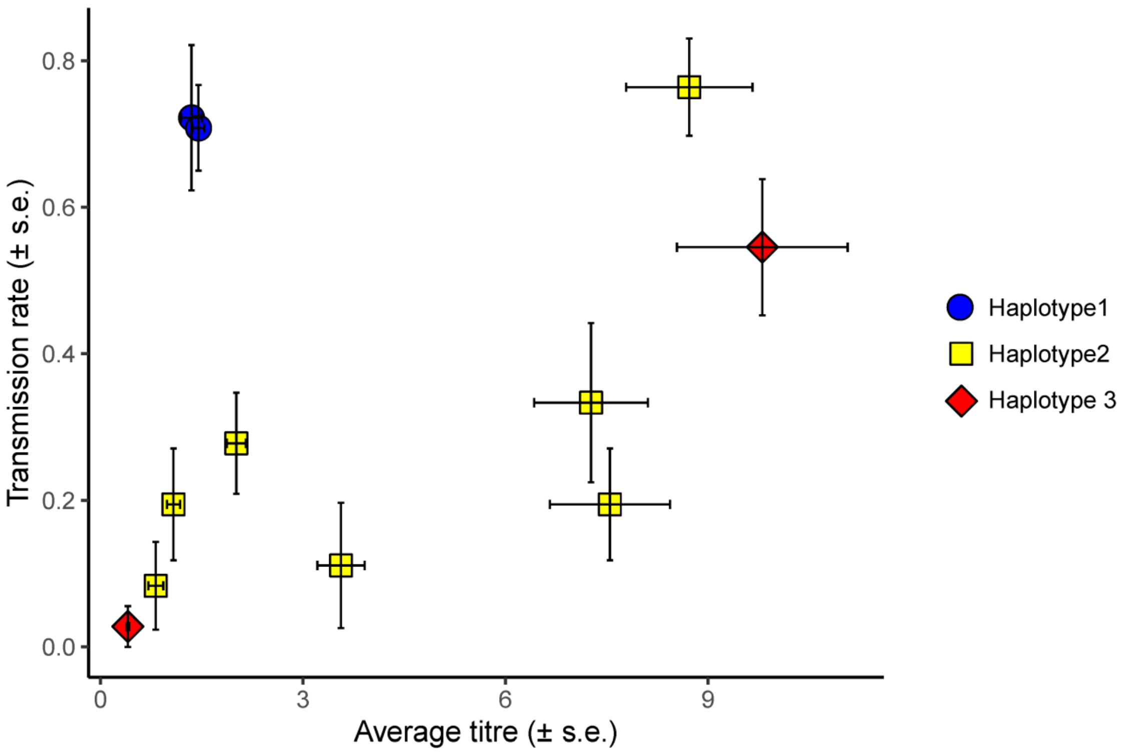

Supplementary Figure 3 – Average titre of different *H. defensa* isolates in donor aphids plotted against the average transmission rate of the isolate without data from aphids of batch 1. Transmission rate corresponds to the number of recipients out of three or four in which a *H. defensa* isolate successfully established after horizontal transmission, i.e. was propagated to the recipient's offspring. In this figure, transmission rate is averaged over three different recipients (37H0, 405H0 and 407H0) and four experimental batches (batch 1 was removed from the analysis). Error bars indicate the standard error and the combination of colour and symbols indicates the haplotype of the *H. defensa* isolate (blue circle = haplotype 1, yellow square = haplotype 2, red diamond = haplotype 3).

Supplementary tables

Supplementary Table 1 – Creation of *H. defensa*-infected A06-407 sublines
For each *H. defensa*-infected subline, the date of creation is indicated. For the clones acting as *H. defensa*-donors, collection date, site and host plant are listed. For each *H. defensa*-isolate, haplotype as determined by sequencing of the two housekeeping genes *murE* and *accD* is indicated.

***Hamiltonella*-infected A06-407 strain**	**Date of creation through microinjection**	**Clone of origin of *Hamiltonella* isolate**	**Collection date of clone of origin**	**Collection site of clone of origin**	**Host plant of clone of origin**	***Hamiltonella* isolate**	***Hamiltonella* haplotype**
407H9	June 2008	A06-09	08.05.2006	La Spezia, Italy	*Vicia faba*	H9	Hap2
407H15	April 2012	A06-15	08.05.2006	Ressora, Italy	*Vicia faba*	H15	Hap3
407H28	April 2012	A08-28	13.05.2008	Altstetten ZH, Switzerland	*Chenopodium album*	H28	Hap2
407H30	March 2009	A06-30	08.05.2006	Sarzana, Italy	*Vicia faba*	H30	Hap2
407H76	March 2009	A06-76	17.05.2006	La Grande Motte, France	*Chenopodium album*	H76	Hap1
407H85	September 2011	A06-85	17.05.2006	Grimaud, France	*Chenopodium album*	H85	Hap3
407H101	September 2011	A06-101	18.05.2006	Le Muy, France	*Vicia faba*	H101	Hap1
407H323	June 2008	A06-323	27.06.2006	Aesch BL, Switzerland	*Vicia faba*	H323	Hap2
407H343	September 2011	A06-343	02.07.2006	Altenhasslau, Germany	*Chenopodium album*	H343	Hap2
407H402	October 2008	A06-402	01.07.2006	St. Margrethen SG, Switzerland	*Chenopodium album*	H402	Hap2
407HAf6	July 2008	Af6	25.05.2004	Zurich, Switzerland	*Euonymus europaeus*	HAf6	Hap2

Supplementary Table 2 – Origin of aphid clones

Collection date, site and host plant for the aphid clones free from *H. defensa.*

Aphid clone	Collection date	Collection site	Host plant	Facultative endosymbiont
A06-37	08.05.2006	Romagna, Italy	*Vicia faba*	none
A06-405	01.07.2006	St. Margrethen SG, Switzerland	*Chenopodium album*	none
A06-407	17.05.2006	St. Margrethen SG, Switzerland	*Chenopodium album*	none
A08-28^{H-}	13.05.2008	Zurich, Switzerland	*Vicia faba*	none, cured from *Hamiltonella defensa* in 2011

Supplementary Table 3 – Overview over experimental procedures

Timetable of the experimental procedures with the day of the procedure in the leftmost row. Days without any procedures are not shown. Black arrows indicate transfer of a set of adults to new plants on four consecutive days.

**1**	Produce grandparents of recipients		Produce parents of donors		Produce grandparents of sentinels
**15**	Produce parents of recipients		Produce donors for (B1)		Produce parents of sentinels
**16**			Produce donors for (B2)		
**17**			Produce donors for (B3)		
**18**			Produce donors for (B4)		
**19**			Produce donors for (B5)		
**27**	Produce recipients (B1)				Produce sentinels (B1)
**28**	Produce recipients (B2)				Produce sentinels (B2)
**29**	Produce recipients (B3)				Produce sentinels (B3)
**30**	Produce recipients (B4)		Transfection (B1)		Produce sentinels (B4)
**31**	Produce recipients (B5)		Transfection (B2)		Produce sentinels (B5)
**32**			Transfection (B3)		
**33**			Transfection (B4)		
**34**			Transfection (B5)		
**36**			Place 4 survivors singly on leaf disc (B1)		Place sentinels on a shared leaf disc (B1)
**37**			Place 3 survivors singly on leaf disc (B2)		Place sentinels on a shared leaf disc (B2)
**38**			Place 3 survivors singly on leaf disc (B3)		Place sentinels on a shared leaf disc (B3)
**39**		Count and discard nymphs (t1) (B1)	Place 3 survivors singly on leaf disc (B4)		Place sentinels on a shared leaf disc (B4)
**40**		Count and discard nymphs (t1) (B2)	Place 3 survivors singly on leaf disc (B5)		Place sentinels on a shared leaf disc (B5)
**41**		Count and discard nymphs (t1) (B3)			
**42**		Count and discard nymphs (t1) (B4)			
**43**		Count and discard nymphs (t1) (B5)<br>Count nymphs (t2) (B1)	Place survivor on new leaf disc (B1)		Harvest adult sentinels (B1)
**44**		Count and discard nymphs (t2) (B2)	Place survivor on new leaf disc (B2)	Discard adult (B1)	Harvest adult sentinels (B2)
**45**		Count and discard nymphs (t2) (B3)	Place survivor on new leaf disc (B3)	Discard adult (B2)	Harvest adult sentinels (B3)
**46**		Count and discard nymphs (t2) (B4)	Place survivor on new leaf disc (B4)	Discard adult (B3)	Harvest adult sentinels (B4)
**47**		Count and discard nymphs (t2) (B5)	Place survivor on new leaf disc (B5)	Discard adult (B4)	Harvest adult sentinels (B5)
**48**				Discard adult (B5)	
**50**			Harvest nymphs (B1)		
**51**			Harvest nymphs (B2)		
**52**			Harvest nymphs (B3)		
**53**			Harvest nymphs (B4)		
**54**			Harvest nymphs (B5)		

Supplementary Table 4 – Polymerase chain reaction conditions
Primers and cycling conditions for PCR reactions to confirm extraction success, presence of *H. defensa* and amplification of genes used for haplotype-typing.

Primers targeting *Hamiltonella defensa*

10F	AGTTTGATCATGGCTCAGATTG
T419/TO419	AAATGGTATTSGCATTTATCG

Primers targeting *Buchnera aphidicola*

16SA1	AGAGTTTGATCMTGGCTCAG
Buch_16S_Afab_R	CTTCTTCGGGTAACGTCAAGAA

Primers for haplotype typing

murE16F	ACTAACGGGAAAACCACTAATAC
murE936R	TTGAGAATGTCAGCGGTAATC
accD291F	TTCTGGAGCACAAAAAGACAC
accD832R	AAGGTTCAGGTTGATGAGTCAG

PCR program for endosymbionts

Heat lid to 95°C

95	°C	3 min	
95	°C	30 sec	10x
65-56	°C	30 sec	
72	°C	60 sec	
95	°C	30 sec	25x
55	°C	30 sec	
72	°C	60 sec	
72	°C	6 min	
10	°C	∞	

PCR program for haplotype testing

Heat lid to 95°C

94	°C	2 min	
94	°C	30 sec	11x
56-46	°C	50 sec	
72	°C	50 sec	
94	°C	30 sec	25x
45	°C	50 sec	
72	°C	60 sec	
72	°C	6 min	
4	°C	∞	

Supplementary Table 5 – Results of a generalised linear mixed effects model for the transmission rate of different H. defensa isolates to different recipients (37H0, 405H0, 407H0). Model predictors were recipient, average titre that an isolate reaches in the donor aphid and haplotype of the isolate (haplotypes 1, 2 and 3). The aphid subline acting as donor during horizontal transmission ('donor') and experimental batch were treated as a random effect. The model did not contain data from batch 1.

	Effect	*LR* χ^2	*df*	*p-value*
Random:	donor	18.76	1	**<0.001**
	batch	2.54	1	**0.111**
Fixed:	titre of *H. defensa* isolate	8.34	1	**0.004**
	haplotype of *H. defensa* isolate	11.48	2	**0.003**
	recipient clone	1.87	2	0.393
	titre : recipient	10.88	2	**0.004**
	recipient : haplotype	10.38	4	**0.034**
